# Paediatric radiotherapy in the United Kingdom: an evolving subspecialty and a paradigm for integrated teamworking in oncology

**DOI:** 10.1093/bjr/tqad028

**Published:** 2023-12-12

**Authors:** Amy Colori, Raymond Ackwerh, Yen-Ch’ing Chang, Kristy Cody, Cathy Dunlea, Jennifer E Gains, Trevor Gaunt, Callum M S Gillies, Claire Hardy, Narinder Lalli, Pei S Lim, Carmen Soto, Mark N Gaze

**Affiliations:** Department of Oncology, University College London Hospitals NHS Foundation Trust, London, NW1 2PG, United Kingdom; Department of Anaesthetics, University College London Hospitals NHS Foundation Trust, London, NW1 2BU, United Kingdom; Department of Oncology, University College London Hospitals NHS Foundation Trust, London, NW1 2PG, United Kingdom; Department of Radiotherapy, University College London Hospitals NHS Foundation Trust, London, NW1 2BU, United Kingdom; Department of Radiotherapy, University College London Hospitals NHS Foundation Trust, London, NW1 2BU, United Kingdom; Department of Oncology, University College London Hospitals NHS Foundation Trust, London, NW1 2PG, United Kingdom; Department of Radiology, University College London Hospitals NHS Foundation Trust, London, NW1 2BU, United Kingdom; Department of Radiotherapy Physics, University College London Hospitals NHS Foundation Trust, London, NW1 2PG, United Kingdom; Department of Radiotherapy, University College London Hospitals NHS Foundation Trust, London, NW1 2BU, United Kingdom; Department of Radiotherapy Physics, University College London Hospitals NHS Foundation Trust, London, NW1 2PG, United Kingdom; Department of Oncology, University College London Hospitals NHS Foundation Trust, London, NW1 2PG, United Kingdom; Department of Paediatric Oncology, University College London Hospitals NHS Foundation Trust, London, NW1 2BU, United Kingdom; Department of Oncology, University College London Hospitals NHS Foundation Trust, London, NW1 2PG, United Kingdom; Department of Oncology, UCL Cancer Institute, University College London, London, WC1E 6DD, United Kingdom

**Keywords:** brachytherapy, children’s cancer, complex photon techniques, multi-professional teamwork, paediatric radiotherapy, proton beam therapy

## Abstract

Many different malignancies occur in children, but overall, cancer in childhood is rare. Survival rates have improved appreciably and are higher compared with most adult tumour types. Treatment schedules evolve as a result of clinical trials and are typically complex and multi-modality, with radiotherapy an integral component of many. Risk stratification in paediatric oncology is increasingly refined, resulting in a more personalized use of radiation. Every available modality of radiation delivery: simple and advanced photon techniques, proton beam therapy, molecular radiotherapy, and brachytherapy, have their place in the treatment of children’s cancers. Radiotherapy is rarely the sole treatment. As local therapy, it is often given before or after surgery, so the involvement of the surgeon is critically important, particularly when brachytherapy is used. Systemic treatment is the standard of care for most paediatric tumour types, concomitant administration of chemotherapy is typical, and immunotherapy has an increasing role. Delivery of radiotherapy is not done by clinical or radiation oncologists alone; play specialists and anaesthetists are required, together with mould room staff, to ensure compliance and immobilization. The support of clinical radiologists is needed to ensure the correct interpretation of imaging for target volume delineation. Physicists and dosimetrists ensure the optimal dose distribution, minimizing exposure of organs at risk. Paediatric oncology doctors, nurses, and a range of allied health professionals are needed for the holistic wrap-around care of the child and family. Radiographers are essential at every step of the way. With increasing complexity comes a need for greater centralization of services.

## Introduction

### Cancer in children and young people

Cancer in children and young people (CYP) is rare. Over 20 years from 1997 to 2016, on average only 3755 cases per year occurred in the United Kingdom in those aged 0-24 years. That comprised 1645 in the true “paediatric” population (0-14 years) and 2110 in “young people” (15-24 years).^1^ Of the 375 000 cancer diagnoses annually in the United Kingdom, only 1% occur in children and teenagers or young adults (TYA), and very much <1% occur in young children.^2^

Cancer in CYP is very diverse, including leukaemias, lymphomas, central nervous system tumours, bone and soft tissue sarcomas, neuroblastic tumours, retinoblastoma, renal tumours, liver tumours, germ cell tumours, malignant epithelial tumours, malignant melanoma, and a range of unspecified cancer types. Their relative incidence varies significantly by age.^1^

Each category is subdivided into many different entities. For example, “renal tumours” encompasses Wilms tumour or nephroblastoma, and mesoblastic nephroma, malignant rhabdoid tumour, clear cell sarcoma of the kidney, and renal cell carcinoma.^3^ Even these are subdivided, for example Wilms tumour is split into favourable and unfavourable histological types, and different disease stages, each requiring a different treatment schedule.^4^

The overall rarity of childhood cancer and wide diversity of subtypes, stages, and risk-groups mean that despite expertise becoming concentrated in increasingly fewer specialist centres, each patient needs careful and individualized multidisciplinary consideration.

This paper describes the current UK service model, and its evolution over time, for the radiotherapy of CYP. It may be used, if desired, as a template for the improvement of paediatric radiation oncology, where necessary, in other countries.

### The development and types of radiation treatment

Though most paediatric malignancies require complex multi-modality treatment, radiotherapy is not always required; its use having declined with improved systemic therapies and more personalized risk stratification.^5^ Currently, one-third of paediatric treatment protocols involve radiotherapy.^6^

“Radiotherapy” includes many different ionizing radiation treatments. In the last century, treatment most commonly used photons, and this modality gradually evolved.^7^ Orthovoltage treatments using surface markings gave way to simple megavoltage treatments (1960-1980); with fields defined on simulator films, planned on a single central-axis slice, as cobalt units and linear accelerators (linacs) became available. In turn, 3D conformal radiotherapy using computerized tomography (CT) planning became the norm from the 1990s onwards. The development of linac technology also allowed the use of electron treatment of different energies. More recently, CT simulators have replaced fluoroscopic X-ray simulators, and advanced photon delivery techniques, including intensity-modulated^8^^,^^9^ and image-guided^10^ approaches, and more sophisticated application of these techniques including adaptive and stereotactic ablative radiotherapy,^11^ have become widely used.

**Figure 1.**
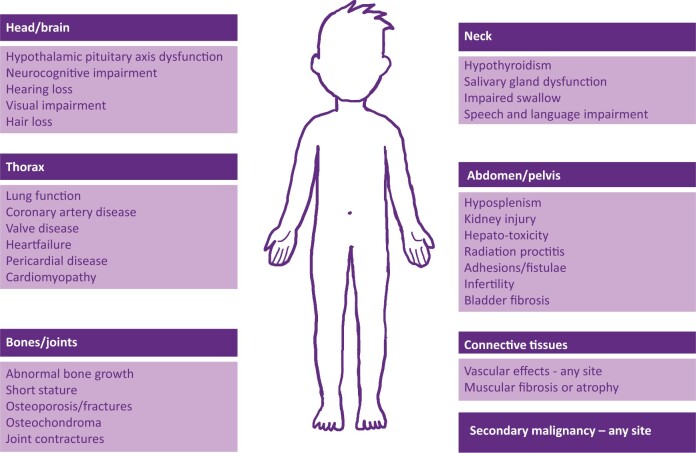
Commonly encountered long-term side effects following radiotherapy in children and young people.

In paediatric radiotherapy, specific indications which were previously considered well-treated by photons are now increasingly treated by proton beam radiotherapy (PBT) as a result of a reduction in the likelihood of late effects predicted by dosimetric studies. It is unlikely that photon versus proton randomized trials will be conducted in paediatric practice to demonstrate that these predictions are correct, but gradually supportive evidence is accumulating. Cure rates are equivalent between modalities, but more favourable dose distributions may offer a substantial reduction in late complications. These include organ dysfunction, impaired childhood development, and, importantly, risk of secondary malignancy (Figure 1). Since 2008, highly selected patients have been referred abroad under UK National Health Service (NHS) commissioning for PBT.^12^ A decade later, the first UK NHS high-energy PBT centre opened in Manchester, followed in 2021 by a second in London. With the increasing availability of PBT, the indications have broadened far beyond the original criteria for the Proton Overseas Programme. The increasing proportion of patients from photon-only centres meeting the criteria to receive PBT has meant a gradual reduction in the numbers of children remaining for treatment in photon centres. NHS England specialist paediatric photon commissioning specifications state that centres must “serve a population sufficient to support a critical mass of infrastructure, such that it can deliver care to children with complex needs and maintain sub-specialist experience, given the heterogeneity of cancer diagnoses in this patient population.”^13^ Maintenance of a quality holistic service in these smaller photon centres has not always been possible so, for example, paediatric radiotherapy is no longer delivered at Aberdeen, Liverpool, or Southampton.

Though external beam radiotherapy predominates, other radiotherapy techniques are used in a small, very carefully selected population, including brachytherapy^14^ and molecular radiotherapy.^15^ Referral of appropriately selected patients to the limited number of national centres with the required expertise and facilities is therefore recommended.^16^

## Patient-centred treatment

### Decision-making in paediatric radiotherapy

Discussion of the management of children with cancer from diagnosis onwards, in a paediatric oncology multi-disciplinary team (MDT) meeting, has been standard practice long before it was mandated in the 2000 NHS Cancer Plan.^17^ Management may also be discussed at tumour site-specific MDTs, and, increasingly, in national advisory groups.^18^ At present, referrals for PBT must be approved by the national proton panel.^19^ Research-focused paediatric clinical oncologists are key MDT members offering awareness of an increasing portfolio of current clinical trials involving radiotherapy.^20^

### The pre-treatment pathway

If radiotherapy may form part of treatment, early interaction with the clinical oncologist and radiotherapy team is recommended.^21^ This provides an opportunity for the child and family to understand radiotherapy as a potential component of treatment, to be introduced to the team, and to receive the required written and verbal information. Lay understanding of radiotherapy is limited,^22^ so an early introduction is valuable.^23^^,^^24^ All necessary information, including factors relevant to the planning, delivery, and subsequent complications of radiotherapy, such as co-morbidity, functional disability, medication and allergies, and social circumstances, will be collated. A detailed radiotherapy referral form, complying with Ionising Radiation (Medical Exposure) Regulations 2018 (IRMER) is completed.^25^

In establishing informed consent, we consider the possibility of significant acute and late effects caused by radiotherapy. Advances in radiotherapy techniques, especially PBT, reduce but do not avoid the risk of late complications.^26^^,^^27^ It is important to discuss the impact of these complications on quality of life and functional outcomes with parents, as well as potential risk-differences between radiotherapy techniques.^28^

In paediatric radiotherapy, consideration of requirements of a form of anaesthesia such as general anaesthesia (GA), or sedation, is also needed. If there is uncertainty (due to age or comorbidity), a dual pathway is followed, aiming for awake treatment, but using GA if necessary.

Regular meetings of the whole radiotherapy team are vital, so that all staff groups are familiarized with the incoming patients, to establish their play specialist needs, anaesthetic requirements, and the relevant considerations for immobilization devices and patient positioning. Clarity about imaging requirements is important for target volume delineation and planning decisions, for example CT scanning levels, choice of MRI sequences to be acquired during planning, use of contrast, and choice of prior diagnostic imaging including CT, MRI, and positron emitted tomography for fusion. Given the complexity of the paediatric planning pathway, we must anticipate individual requirements ahead of time to avoid treatment delays which would result from having to repeat any of the planning stages. This aligns with the NHS England policy of *Getting It Right First Time* (GIRFT), a national programme designed to improve the treatment and care of patients through in-depth review of services, benchmarking, and presenting a data-driven evidence base to support change.^29^ With the evolution of more precise and conformal radiotherapy techniques with tighter margins, pre-treatment planning decisions must consider potential internal organ motion, to avoid the risk of geographical miss.^30^

Once scanned, the clinical oncologist delineates the target volumes and organs at risk (OAR) and prescribes treatment. International guidance now aims to standardize OAR delineation.^31^ There should be an adequately detailed, explanatory “planning communication document” to enable physicists and dosimetrists to understand what is required.

Peer review of target volumes is universally mandated by the Royal College of Radiologists.^32^ Additional external peer review for quality assurance (QA) in paediatric radiotherapy clinical trials, though the European Society of Paediatric Oncology (SIOP-E), *Quality and Excellence in Radiotherapy and Imaging for Children and Adolescents with Cancer across Europe in Clinical Trials* (QUARTET) project is often required.^33^

Physicists and dosimetrists will then prepare a treatment delivery “plan” which optimally provides full, even coverage of the target volume to the prescribed dose, while minimizing dose to the OARs, respecting maximum dose constraints to reduce the incidence of late effects wherever possible. In PBT, planning may be more complex, and a range of uncertainties is taken into account to provide robust optimization.^34^

However, sometimes compromises are necessary where full intended treatment dose might result in excessive dose to an OAR. In this situation a judgement is required as to whether it is better to deliver the full dose and risk damage to the OAR, or to keep the OAR dose safe and risk a lower tumour control probability. Determining the optimal plan requires peer-review by both clinical oncologists and physicists.^35^

Developing tissues in young children can be more sensitive to radiation-induced late effects than the corresponding organs in adults, but paediatric dose-constraints are not well-established. The *Pediatric Normal Tissue Effects in the Clinic* (PENTEC) collaboration is addressing this with a series of comprehensive reviews.^36^ The risks of even relatively low-dose radiotherapy are increasingly being recognized.^37^ The International Late Effects of Childhood Cancer Guideline Harmonization Group is providing evidence-based advice about late effects which will be of value in minimizing morbidity in the future.^38^

### Treatment delivery

Once planned, prescribed, quality-assured, and approved; delivery of treatment can commence. The patient’s daily experience will vary depending on whether treatment is given awake or under GA. Daily positional checks are required before each fraction. Image-guidance is now the norm, with 2D kilovoltage (kV) imaging for set up, and 3D cone beam CT (CBCT) to ensure accuracy of plan delivery, stability of patient parameters such as external contour or OAR filling and patient positioning in cases where kV imaging is insufficient.^39^

Regular image review, comparing CBCT and planning CT, determines the need for replanning. Indeed, changes in tumour volume may be an indication for adaptive radiotherapy.^40^^,^^41^ Potentially surface guidance may be of value in monitoring changes.^42^ Use of motion management, such as breathing gated techniques or Deep Inspiration Breath-Hold is feasible, and may reduce OAR irradiation (especially heart and lung).^43^^,^^44^ Relative to photon radiotherapy, accuracy of PBT dose delivery is especially sensitive to changes in body contour due to weight loss or gain, positioning, and variations in abdomino-pelvic organ contents.^45^

Careful clinical monitoring of children on treatment for acute toxicity, nutritional stability, nausea and vomiting, myelosuppression and infection, and physiotherapy/occupational therapy needs is required. Optimal toxicity management allows children to maintain normality during radiotherapy, including attending school, or hospital school if not living locally.

### After treatment

Close follow-up occurs until acute treatment reactions subside. There may be planned continuing treatment with surgery and/or chemotherapy. Follow-up with imaging may be required to assess treatment response, and for surveillance. Assuming there is no recurrence, long-term follow-up in a multidisciplinary late effects clinic is required for late morbidity (Figure 1), which should be carefully documented.^46^^,^^47^ For PBT, this is collected as part of the commissioners’ outcome programme.^48^ This includes surveillance when there is a high risk of radiation-induced second cancers.^49^ Provision of a “survivorship passport” may be of value.^50^

## Multi-professional delivery of radiotherapy for children

### The paediatric radiotherapy team

The success of the treatment pathways outlined above depends on the involvement of a wide range of different professions and medical disciplines, all with additional training in the field of paediatric radiotherapy. See Table 1 for a glossary of roles. However, currently, no UK medical, nursing or allied health professional training scheme offers a specific, recognized, paediatric radiotherapy qualification. The Paediatric Radiation Oncology Society promotes learning opportunities in this field,^51^ and other professional bodies provide continuing professional development programmes. Here we consider the specialized contributions of these different staff groups. In addition, management of these children may also require the input of dieticians, speech and language therapists, physiotherapists occupational therapists, and psychologists. Long-term follow-up is also multi-professional, including monitoring of late effects by endocrinology, audiology, ophthalmology, and other specialists.

**Table 1.** Glossary of terms.

Health play specialist	Health play specialists work with children and young people when they access healthcare services in different healthcare settings using play as the way to build relationships with children and help them have the best possible experience during a hospital visit or procedure.
Clinical nurse specialist	Advanced nurse providing advice and treatment to patients in a specialized setting.
Paediatric anaesthetist	Specialized doctor responsible for providing anaesthesia to children during operations and procedures.
Paediatric therapeutic radiographer	Therapeutic radiographers are responsible for the *planning* and *delivery* of accurate radiotherapy treatments to children. Specialist radiographers may be the key worker coordinating care, and the first point of contact for families.
Paediatric oncologist	A paediatrician specialized in the treatment of children with cancer, including the use of systemic anti-cancer therapy including chemotherapy and immunotherapy.
Medical physicist	Radiotherapy medical physicists are clinical scientists responsible for planning and evaluation of radiotherapy plans, quality assurance, and radiation protection.
Dosimetrist	Medical professional who is certified to develop radiotherapy treatment plans and to calculate and deliver doses of radiation to cancer patients.
Clinical (Radiation) oncologist	Specialist doctor trained to deliver both radiotherapy (and systemic anti-cancer therapy in the adult population).

### Play specialists

**Figure 2.**
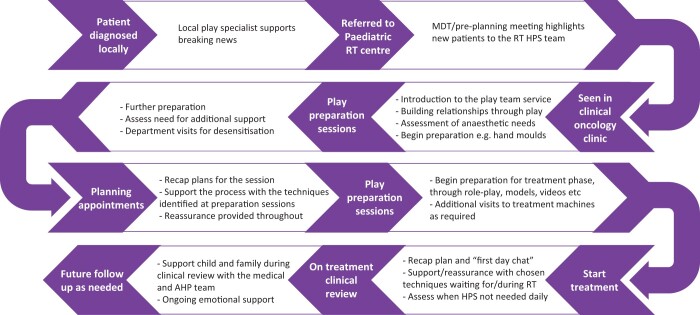
The health play specialist is involved in different ways throughout the child’s pathway through radiotherapy planning and treatment. Abbreviations: HPS = health play specialist; MDT = multidisciplinary team; RT = radiotherapy.

**Figure 3.**
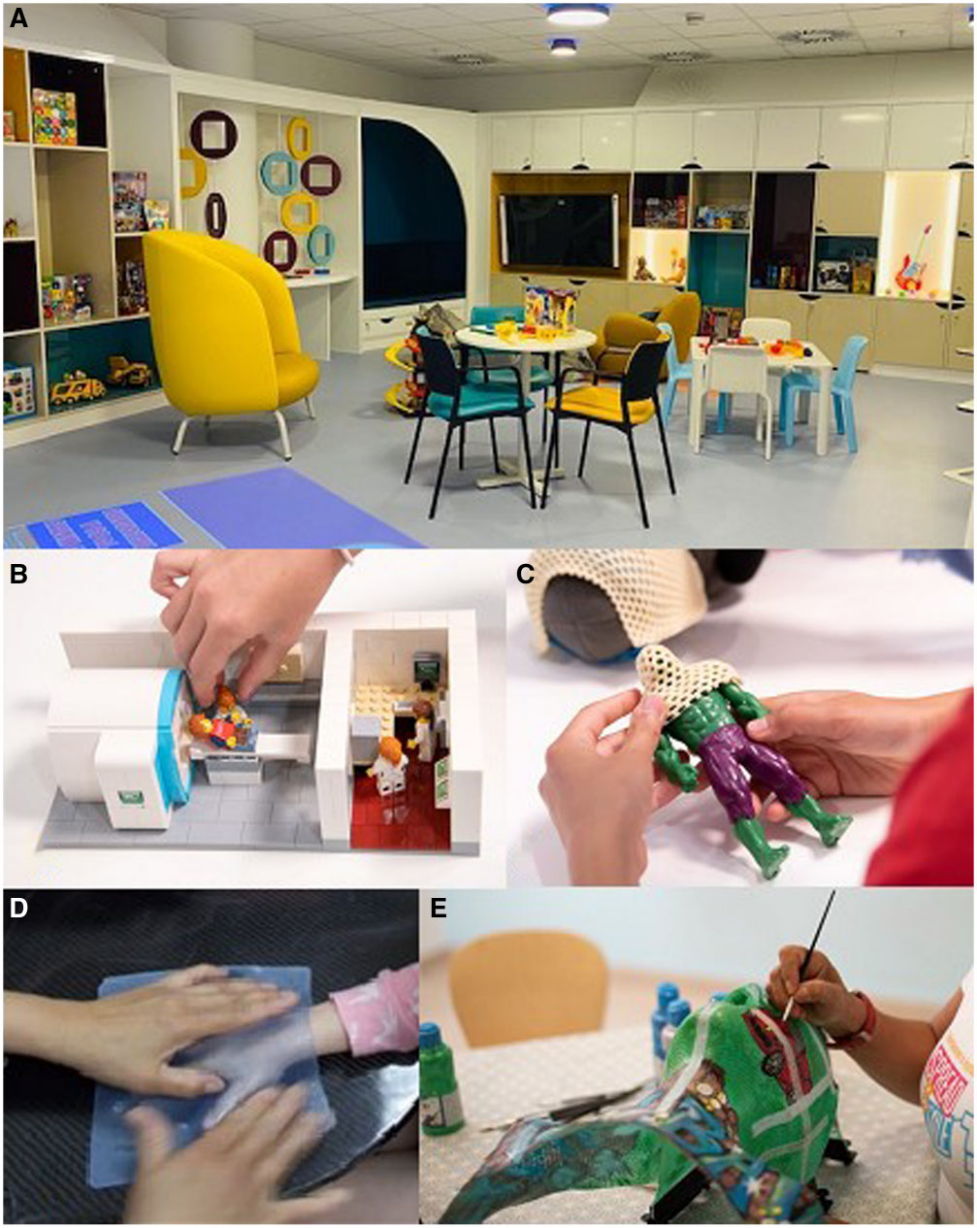
The play room (A) is the epicentre of the health play specialists work, using models of equipment (B) to familiarize children with planned procedures, demonstrating the use of immobilization devices on toys (C) and making hand moulds (D) to let children experience the warmth and feel of thermoplastic before making a head shell, which can be decorated as the child chooses (E).

There are many components of the role of the health play specialist (HPS) within the paediatric radiotherapy pathway (Figure 2), but involvement from referral in both the clinic and the play room (Figure 3A) is key to building a trusting relationship and helping to support the patient and family though each step.^52^ Working with children has many challenges and HPS must be creative in the ways in which they engage individual children and deliver age-appropriate information.^53^ By assessing the child’s ability to cope with procedures at an early stage, play specialists provide the vital role of establishing whether radiotherapy can be delivered without the need for GA.^54^

Several aspects of radiotherapy can elicit fear and anxiety, including immobilization devices and tattoos. Play specialists share information appropriate to the child’s age and developmental needs.^55^ using resources such as photo preparation books, videos, and models of CT and MRI scanners (Figure 3B) and linacs which allow children to role play using their favourite characters (Figure 3C).

Play sessions familiarize patients with the real equipment to be used, and the noises, movements and people carrying out procedures. Making a practice hand (Figure 3D) mould allows the child to experience the warmth of thermoplastic to desensitize them before making the definitive shell.^56^

Many resources for distraction, such as music, projected movies, talking or reading a story over a sound system may help to mitigate the length of the procedure.^57^ Giving choices where possible allows the child some opportunity for control. This can include choosing their toys, blanket, or gown, how their mask is decorated (Figure 3E), what they will listen to, or who will be in the room with them during radiotherapy preparation.

### Paediatric anaesthetic team

Children <3 years of age, or 5 if requiring a head shell for immobilization, and some older children with anxiety or neurocognitive disorders, are not able to undergo conventional radiotherapy awake.^58^ Since PBT takes longer to deliver, GA may be required more often in older children. Therefore, an anaesthetic team that is suitably trained for the challenges of both paediatric and remote site anaesthesia, is vital. This is accomplished by administering either sedation, where the patient maintains their own natural airway, or GA, where an artificial airway (laryngeal mask airway [LMA] or endotracheal tube) is inserted. Anaesthetics teams can also provide additional regional anaesthesia as needed, such as caudal blocks in children undergoing brachytherapy for genito-urinary tumours.

A key challenge for anaesthetists, given the aim of completing treatment without any unplanned interruptions, is how to proceed at times when sedation or anaesthesia would not usually be administered, such as concurrent upper respiratory tract illness. In such cases, full inter-speciality discussion takes place. Ultimately, if a child is unsafe to undergo GA, radiotherapy treatment is missed that day and compensated where possible.^59^

There is higher risk of critical events during sedation and anaesthesia in young children.^60–62^ Additionally, the effects of cancer, prior surgery, and chemotherapy, together with concomitant acute illness, increase the risk. Furthermore, potential radiotherapy-related airway swelling means that careful pre-procedural assessment is extremely important. Patient optimization for anaesthesia includes: play therapy for anxiety, anti-emetic therapy, naso-gastric tubes, and fluids, antibiotics and secretion management where required.

Further to normal GA practice, an additional, unique, consideration for the radiotherapy anaesthetist is obtaining optimum positioning for planning scans and then consistently reproducing this throughout treatment. Anaesthetists ensure children have long-term intravenous access in-situ before starting a course of daily GA. While deep sedation may be most commonly used for radiotherapy worldwide,^63^ there remains no consensus on the best anaesthetic technique. Encouragingly, the incidence of adverse incidents is very low (<1%) over the full range of drugs used for sedation and anaesthesia.^64^^,^^65^

The choice is further influenced by maintenance of physiological homeostasis.^66^ Normal oxygen and carbon dioxide levels are easier to maintain under GA, with an LMA and mechanical ventilation. Furthermore, the risk with deep sedation, particularly with airway swelling and/or a tight immobilization shell, is that further airway obstruction may result in the need to convert to GA, precipitating the need to replan treatment.

Patients generally recover well with modern sedative and anaesthetic agents, allowing early recommencement of feeding facilitating weight maintenance. The recovery period is facilitated by dedicated paediatric recovery and ward nurses, expert in creating a positive patient experience throughout daily therapy.

### Nursing

The role of the clinical nurse specialist (CNS) in cancer services generally is well established, indeed, all patients should be allocated a named CNS for input and support immediately following diagnosis.^67^ Patients with an allocated CNS are more positive about their experience of care, with their CNS being regarded a valuable person in decision-making and a trusted source of information.^68^ Indeed, evidence suggests that adult patients without a named CNS may have worse outcomes.^69^ Given the equivalence of the roles in the 2 populations, this may well apply to children too.

**Figure 4.**
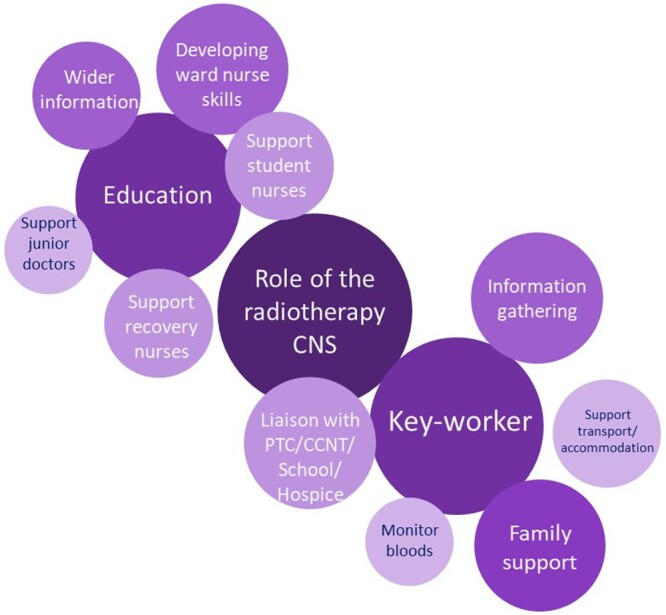
The paediatric radiotherapy clinical nurse specialist is the child’s key worker and has many responsibilities. Abbreviations: CNS = clinical nurse specialist; CCNT = community children’s nursing team; PTC = principal treatment centre.

The paediatric radiotherapy CNS is the child’s key worker (Figure 4), supporting the family as a named, trusted, single point of contact. They have immediate access to the whole health professional team, facilitating timely input. Simple mobile phone messaging tools make contact easy for parents. With the centralization of paediatric radiotherapy, families find themselves away from home for weeks. Here, the CNS is especially important to help the family to navigate an unfamiliar hospital and care team. The impact of separating a young person from their home support networks for up to 6 weeks is considerable. In addition to healthcare liaison, the CNS links to their home school, supports access to the hospital school, and facilitates local enjoyable experiences and home visits, where possible.

In addition to enhancing patient experience, the cancer CNS will often be the first healthcare provider to assess the patient.^70^ On completion of radiotherapy, the CNS will liaise with the local community children’s nursing team and social work team to ensure a full psychosocial and medical handover, ensuring smooth transition and continuity of care.

### Radiologists

Imaging is central to the management of paediatric patients with both central nervous system and other malignancies, from initial diagnosis, staging, surgical and radiotherapeutic planning, to assessing treatment response and surveillance thereafter.

A broad array of independent and hybrid diagnostic and functional modalities are established in diagnostic practice, and these are well covered within the literature.^71–74^ However, the involvement of the clinical radiologist in the planning of radiotherapy for children is becoming more common place, as radiotherapy imaging and delivery becomes more sophisticated.

Discussion of imaging with clinical radiologists is invaluable and has been shown to change volume delineation in up to 35.6% of cases (also increasing the reported confidence of the responsible clinical oncologist).^75^

Regular meetings between paediatric radiologists and neuroradiologists, clinical oncologists, and pre-treatment radiographers, facilitates the optimal sequence selection and field of view coverage at the pre-planning stage. Such clinical radiology involvement is particularly important in the era of high-dose highly conformal radiotherapy and PBT, to accurately define the target and avoid OAR.^76^ This is paramount in children, given the risk of late effects of therapy and radiation-induced secondary cancers.

### Paediatric oncologists

Paediatric oncologists in the United Kingdom are paediatricians who specialize in the care of children with cancer along the whole of the patient journey. For CYP being treated with radiotherapy, their role includes: delivering chemotherapy and increasingly immunotherapy; managing treatment-related toxicities; and addressing general paediatric issues, including safeguarding if concerns are raised. Paediatric oncologists co-ordinate the wider supportive care MDT, including occupational therapists, physiotherapists, speech and language therapists and dieticians, to care for patients throughout their treatment. Close liaison with both the CYP Principal Treatment Centre (PTC), and Paediatric Oncology Shared Care Unit (POSCU) who support the child and family locally, is essential.

The safe delivery of concurrent chemoradiotherapy requires detailed understanding of the treatment pathway to date, especially any toxicities. Clinical trial treatments, and off-trial guidelines can change rapidly, so clinicians need to keep up-to-date via national networks. Doses may need to be adjusted or omitted based on previous toxicities or risks of administration alongside radiotherapy; and further chemotherapy-related toxicity may develop during radiotherapy treatment. So prescribing and administering chemotherapy during radiotherapy is a careful balancing act between aiming to deliver treatment as intended, while minimizing acute and long-term toxicities. Therefore, concurrent chemotherapy should only be delivered in centres who have this necessary paediatric expertise and understanding.

During treatment, teams proactively provide supportive care including blood product support, prophylactic antibiotics, nutritional support, pain relief—as well as management of acute toxicities such as febrile neutropaenia. Anticipating the impact of previous treatments (such as myelosuppression or chemotherapy-induced nausea and vomiting) can help tailor supportive care to the needs of the individual child, improving the patient experience.

Mutual understanding of the context and radiotherapeutic intent informs the emotional support that families receive from the paediatric oncology team. Reassurance can be provided about what can be expected. Additionally, the team have experience of supporting families through palliation and end-of-life care.

### Physicists and dosimetrists

Medical physicists and dosimetrists work in unison to ensure that the required amount of radiation is delivered to the tumour, minimizing dose elsewhere. This fundamental principle of IRMER regulations underwrites innovation of new treatment technologies and techniques, which increase accuracy and precision.^25^ While dosimetrists focus on the technical design and delivery of radiotherapy plans, medical physicists employ their biophysical knowledge to ensure to the quality of these plans and the equipment delivering them.

Reducing unnecessary dose is especially critical for young patients, lowering chance of secondary malignancy and late side-effects.^77^ Evolution of radiotherapy techniques has allowed continuous refinement of radiotherapy planning practice, facilitating ever more conformal dose distributions and OAR sparing.

However, conformality comes at a cost, and this has particular relevance in the paediatric population. In PBT, dose distributions have steeper fall off. So for example in craniospinal irradiation, we must ensure the balance of adequate gross tumour volume and clinical target volume coverage with homogenous vertebral dose to enable symmetric bone growth.^78^^,^^79^

Medical physicists conduct extensive QA programmes to ensure equipment is performing to the highest specifications and that the margins we use to ensure tumour coverage are appropriate. With this quantification of uncertainty, dose to neighbouring OAR can be reduced with confidence. Close collaboration between dosimetrists, physicists, and clinical oncologists facilitates planning decisions for the patient at hand and informs future considerations.

A final factor that has become increasingly critical, with tighter margins, is image guidance. Ensuring that the dosimetry is robust to any potential changes during treatment set, especially in modalities like PBT is paramount to ensuring safe and effective delivery. This means increasing involvement of the physics and dosimetry team in the on-treatment phase of the patient’s radiotherapy journey. Real-world data show this is vital. All these developments and improvements require more and more of a close working relationship between all disciplines throughout the treatment and ultimately results in better outcomes for patients.

### Therapeutic radiographers

Therapeutic radiographers with paediatric expertise are central to the effective delivery of radiotherapy to CYP. Radiographers fulfil various roles within the patient pathway, including: evaluating and making immobilization for treatment, the acquisition of planning scans, image registration for volume delineation, OAR delineation, field placement in palliative radiotherapy, and treatment delivery. Radiographers acquire regular kV and CBCT images, which they evaluate to ensure the accurate delivery of external beam therapy. Having trained predominantly in the adult setting, they must develop their skills to achieve all this in the paediatric population.

Specialism can lead to a key facilitator role within the MDT, where senior radiographers provide direct support and information to patients and families, undertake on-treatment reviews, lead in research and technique development, while offering expertise to the wider MDT regarding patient pathways, which is essential for the delivery of services to CYP.

### Clinical oncologists

Clinical oncologists begin training in general adult medicine and subsequently become accredited in the treatment of cancer with radiation and systemic therapies. Despite the lack of a specific paediatric training, they have developed subspecialist expertise in the various forms of radiotherapy for cancer in CYP. Unlike tumour site specializations within clinical oncology, paediatric radiotherapy is anatomically unconstrained, and it also utilizes all forms of radiation treatment. There is therefore a huge diversity of practice, and scope for subspecialization, even within paediatric radiotherapy, in larger departments.

Though they lead the paediatric radiotherapy team, clinical oncologists cannot provide radiotherapy treatments alone, and they act as the conductor of an orchestra of therapy radiographers, physicists, dosimetrists, play specialists, anaesthetists, paediatric oncology doctors, and nurses. While ensuring delivery of the best clinical care is their primary role, they often have extensive research, education and management commitments.

## Conclusions

Radiotherapy is a vital component of many CYP treatment schedules. Harmonious working of the various professionals involved in its delivery, determines its success. Guiding our children and TYA through the radiotherapy pathway requires intensive multidisciplinary clinical input. Arguably, there are few other clinical settings in which such an involved and integrated approach is as important.

Given the rarity of childhood cancers, proactive enrolment of patients in clinical trials is an integral part of the pathway. Such research has already done so much to improve outcomes and will continue to do so.

With a wide range of treatment strategies, close attention must be paid to determining the right treatment at the right time. Crucially, radiotherapy is routinely held back in very young children, to allow their growing tissues which may be more susceptible to radiation to develop. As toxicity data accumulates, understanding of the timing and impact of radiotherapy will improve.

The landscape of UK paediatric radiotherapy delivery is changing. NHS commissioned indications for PBT are widening, meaning more CYP will be treated in a national PBT centre. This promises fewer late effects, but carries the social impact of up to 6 weeks of treatment, far from home. While centralization of paediatric clinical oncology expertise has significant benefits, the loss of local pathways, might deprive patients, if they are too unwell to travel, of palliative treatments. Whilst we have increasingly sophisticated radiotherapy techniques at our disposal, their best use can only be decided following the involvement of the paediatric radiotherapy team in its entirety.
